# Prevalence of sleep disorders and daytime sleepiness depends on many influencing factors that should be taken into account

**DOI:** 10.1590/1806-9282.20240635

**Published:** 2024-09-02

**Authors:** Josef Finsterer

**Affiliations:** 1 Neurology and Neurophysiology Center – Vienna, Austria.

Dear Editor,

We read with interest Souza et al.'s article on a cross-sectional study of sleep quality and daytime sleepiness in 179 students at a private university between August 2021 and March 2022 using the Pittsburgh Sleeping Quality Index (PSQI) and the Epworth Sleepiness Scale (ESS) in the form of an electronic questionnaire^1^. According to the PSQI, 65% of students had poor sleep quality, with no difference between gender, BMI, and graduation category^1^. Students with a BMI >25 kg/m^2^ had longer sleep latency and shorter sleep duration than students with a lower BMI^1^. According to ESS, 44% of students had daytime sleepiness^1^. The study is impressive, but some points require discussion.

The first point is the use of an electronic questionnaire. Electronic questionnaires have the disadvantage that the correctness of the answers cannot be easily checked, and it is difficult to judge whether the addressee is actually the person who answered and whether the respondent was mentally capable of understanding the questions correctly and giving appropriate answers.

The second point is that various causes of sleep loss and daytime sleepiness were not taken into account. Sleep disorders can also be due to unusually high consumption of adrenergic stimulants (coffee, tea, cacao, cola, Red Bull, nicotine), acute or chronic stress, abuse of illegal drugs, alcohol addiction, unpleasant sleeping places (bright, noisy, increased humidity, not well tempered), unusually frequent use of electronic devices (laptop, tablet, cell phone), abuse of TV watching or computer work before going to bed, chronic cardiac disease, chronic muscle disease, endocrine disease, metabolic disease, and psychiatric disorder. Furthermore, it would have been mandatory to report the current medication of all included patients. We should also know how many of the students included were using illegal drugs, either stimulants (e.g., speed) or sedatives (e.g., cannabis).

A third point is that the study was obviously carried out during the pandemic. Therefore, it is imperative to rule out complications of SARS-CoV-3 infection^2^ or SARS-CoV-2 vaccination^3^, including post-COVID-19 syndrome or long post-COVID-19 vaccination syndrome. We should know how many suffered from the SARS-CoV-2 infection and how many suffered from severe side effects after the SARS-CoV-2 vaccination.

A fourth point is that the number of females was significantly higher than that of men. This inequality can lead to bias.

A fifth point is that sleep quality can also depend on a person's social status and income. We should know whether the cohort could be divided into high- and low-income students and whether sleep quality and daytime sleepiness differed between high- and low-income students. It should also be reported whether academic performance differs between people with and without sleep disorders or daytime sleepiness.

In summary, the excellent study has limitations that make the results difficult to interpret. Removing these limitations could strengthen and support the study's message. Sleep quality and daytime sleepiness depend on numerous influencing factors that must be included in the analysis in order to make conclusive statements.
